# Medical Ethics

**Published:** 1847-09

**Authors:** 


### EDITORIAL DEPARTMENT.

*Medical Ethics.*—We present to our readers in the eclectic department of this No., the Code of Medical Ethics adopted by the late Medical Convention. Presuming that it will be generally considered satisfactory, if, indeed it be not all that could be desired, we trust that it will be formally accepted by state and local associations throughout the country. Great advantage will accrue from the adoption of codified rules by general agreement, so that there may be entire uniformity in the ethical laws by which the profession shall profess to be governed. The code submitted by the convention to the profession of the United States, has been compiled and drawn up with care, and perhaps could not be materially improved. Let it then be received as the standard system, and let all who have at heart the honor and usefulness of the profession, make it an object, not only to regulate their own conduct in strict conformity with its requirements, but to use all proper, judicious means to secure from others rigid compliance with its behests.

It must be evident to any one who has betowed reflection on the subject, that one of the most effective methods of advancing the position of the medical profession both in the respect of medical men themselves, and in the confidence of the public, is for its members to maintain in all their relations, principles of integrity and honor. Complaint is frequently made of the lowness of estimation in which the profession is held by some: but why is it so? It is due, in no small degree, to the impressions resulting from the non-observance of medical ethics by unworthy members of the profession. When people perceive that regularly recognized members of the profession have no regard for their brethren, and are unscrupulous in their efforts to promote their own selfish purposes, is it surprising that the profession fails to receive that consideration which it ought to claim and preserve? It is not for the protection of the private interests of honorable members of the profession that the enforcement of medical ethics makes its strongest appeal, but it is in this way only that the profession itself can be respectable and respected. We would urge upon local associations every where, (and for this, if for no other purpose, societies should be formed in places where they do not exist) after adopting this as the standard code, to take proper cognizance and action upon every violation of it. Let a condition of continued recognition and fellowship be conformity to the letter and spirit of the code. Let every society not hesitate, nor neglect to act promptly and decidedly on any known infringement by whomsoever committed; and whenever it be deemed just and necessary to dissolve all connection with those who are incorrigibly reckless of its rules, let measures be taken to apprise both the profession and public of the fact. In this way lines distinct and broad will be drawn between the worthy and unworthy members of the profession; the latter will cease to be held responsible for, and participate in the odium of the disreputable conduct of the former; and the character of the profession will be secure against one of the most powerful causes of its depreciation in public sentiment. There is much to be said on this important subject, and we may be tempted to resume it in a future No.

